# Dose evaluation indices for total body irradiation using TomoDirect with different numbers of ports: A comparison with the TomoHelical method

**DOI:** 10.1002/acm2.12540

**Published:** 2019-02-05

**Authors:** Yuki Kasai, Yukihide Fukuyama, Hiromi Terashima, Katsumasa Nakamura, Tomonari Sasaki

**Affiliations:** ^1^ Department of Health Sciences Graduate School of Medical Sciences Kyushu University 3‐1‐1 Maidashi, Higashi‐ku Fukuoka City Fukuoka 812‐8582 Japan; ^2^ Department of Radiology Harasanshin Hospital 1‐8 Taihakumachi, Hakata‐ku Fukuoka City Fukuoka 812‐0033 Japan; ^3^ Department of Radiation Oncology Hamamatsu University School of Medicine 1‐20‐1 Handayama, Higashi‐ku Hamamatsu City Shizuoka 431‐3192 Japan; ^4^ Department of Health Sciences Faculty of Medical Sciences Kyushu University 3‐1‐1 Maidashi, Higashi‐ku Fukuoka City Fukuoka 812‐8582 Japan

**Keywords:** TomoDirect, TomoHelical, TomoTherapy, total body irradiation

## Abstract

TomoDirect has been reported to have some advantages over TomoHelical in delivering total body irradiation (TBI). This study aimed to investigate the relationships between the number of ports and the dose evaluation indices in low‐dose TBI in TomoDirect mode using 2–12 ports and to compare these data with those for the TomoHelical mode in a simulation study. Thirteen patients underwent low‐dose TBI in TomoHelical mode from June 2015 to June 2016. We used the same computed tomography data sets for these patients to create new treatment plans for upper‐body parts using TomoDirect mode with 2–12 beam angles as well as TomoHelical mode. The prescription was 4 Gy in two equal fractions. For the TomoDirect data, we generated plans with 2–12 ports with approximately equally spaced angles; the modulation factor, field width, and pitch were 2.0, 5.0 cm, and 0.500, respectively. For the TomoHelical plans, the modulation factor, field width, and pitch were 2.0, 5.0 cm, and 0.397, respectively. D2, D98, D50, and the homogeneity index (HI) were evaluated to compare TomoDirect plans having 2–12 ports with the TomoHelical plan. Using TomoDirect plans, D2 with four ports or fewer, D98 with 10 ports or fewer, D50 with four ports or fewer and HI with five ports or fewer showed statistically significantly worse results than the TomoHelical plan. With the TomoDirect plans, D2 with seven ports or more, D50 with eight ports or more, and HI with eight ports or more showed statistically significant improvement compared with the TomoHelical plan. All of the dose evaluation indices of the TomoDirect plans showed a tendency to improve as the number of ports increased. TomoDirect plans showed statistically significant improvement of D2, D50, and HI compared with the TomoHelical plan. Therefore, we conclude that TomoDirect can provide better dose distribution in low‐dose TBI with TomoTherapy.

## 1 INTRODUCTION

Total body irradiation (TBI) is widely used in conjunction with chemotherapy as a part of the conditioning regimen of hematopoietic stem cell transplantation (HSCT) for hematologic malignancies.^1^, ^2^, ^3^, ^4^, ^5^, ^6^ The most commonly applied total dose and fractionation schedule of myeloablative TBI is 12 Gy, twice daily, over 3 days.^7^ Low‐dose TBI has appeared as an effective form of conditioning in reduced‐intensity HSCT for patients who cannot tolerate myeloablation because of their age or comorbidity. The prescription dose of low‐dose TBI ranges from 2 to 8 Gy in one to four fractions.^7^, ^8^, ^9^, ^10^

TomoTherapy (Accuray, CA, USA) is the delivery of intensity‐modulated radiation therapy using the rotational delivery of a fan beam in the manner of a computed tomography (CT) scanner.^11^ Currently, TomoTherapy has two radiation modes, TomoHelical and TomoDirect. The former delivers treatment with 360° gantry rotation while the couch is translating through the gantry. In contrast, the latter delivers treatment with 2–12 predetermined, discrete angles using a fixed gantry as the couch passes through.^1^, ^12^, ^13^, ^14^

Some groups have reported the roles and feasibility of TBI using the TomoHelical mode; these studies also showed that TomoHelical offers advantages compared with conventional linear accelerator‐based approaches in terms of dose homogeneity at the target lesion and organ at risk (OAR) sparing.^3^, ^4^, ^5^, ^6^, ^15^, ^16^, ^17^ There are few reports of TBI using TomoDirect; however, Salz et al.^1^ investigated TBI using TomoDirect with 12 static ports with equally spaced angles. They reported the potential advantages of TomoDirect over TomoHelical in TBI as follows. (a) It decreases the risk of interstitial pneumonitis because TomoDirect uses a maximum of 12 fields; since the lung irradiation time from the beginning to the end of treatment is extended, the average dose rate of the lung seems to be decreased. (b) Beam expansion on both edges by a maximum of five leaves each (3.125 cm at the isocenter) if the leaves on the edge of the multi‐leaf collimator (MLC) are not used. Therefore, even if a set‐up error up to 2 cm of the surface occurs, sufficient dose distribution is ensured. (c) The dose heterogeneity in the circulating blood is improved.^1^, ^18^

As far as we know, no report investigating the effect of the number of ports on the dose evaluation indices in TBI using TomoDirect has been published. In this study, we report the relationships between the number of ports and the dose evaluation indices in low‐dose TBI with TomoDirect using 2–12 ports and compare the data with those for TomoHelical in a simulation study.

## 2 MATERIALS AND METHODS

### 2.1 Patients

Thirteen patients underwent irradiation with 4 Gy in two equal fractions of TBI using the TomoHelical mode of TomoTherapy from June 2015 to June 2016 at Harasanshin Hospital. In this study, we only investigated patients who underwent low‐dose TBI because there were too few cases of full‐dose TBI (12 Gy/6 fractions) for myeloablative HSCT at our institution. We used the same CT data sets to replan using the modes of TomoHelical and TomoDirect with 2–12 beam angles for this study. The patient characteristics are shown in Table 1. The patients included 10 males and three females with ages ranging from 30 to 72 yr (median, 63 yr).

**Table 1:** Patient characteristics

Patient no.	Age (yr)	Sex	Diagnosis	Height (cm)	Weight (kg)	BMI	Gantry period (s)	
							Pitch = 0.397	Pitch = 0.430
1	65	M	ALL	159	51	20.2	20.4	21.7
2	60	M	DLBCL	163	38	14.3	20.0	20.1
3	44	M	MDS	165	52	19.1	20.2	21.3
4	68	M	ALL	169	62	21.7	20.6	21.8
5	30	M	AA	172	54	18.3	20.0	21.1
6	68	M	AML	166	50	18.1	20.0	21.3
7	58	M	AML	159	47	18.6	20.2	21.3
8	53	M	AML	175	63	20.6	21.1	22.1
9	72	F	ATLL	150	51	22.7	20.0	21.0
10	39	M	AA	162	55	21.0	21.2	22.5
11	64	M	CMML	170	53	18.3	20.9	22.1
12	65	F	ALL	150	45	20.0	20.3	21.5
13	63	F	FL	146	39	18.3	20.1	21.2

M: male; F: female; ALL: acute lymphoid leukemia; DLBCL: diffuse large B‐cell lymphoma; MDS: myelodysplastic syndromes; AA: aplastic anemia; AML: acute myelogenous leukemia; ATLL: adult T cell leukemia/lymphoma; CMML: chronic myelomonocytic leukemia; FL: follicular lymphoma; BMI: body mass index [=weight (kg)/height^2^ (m)].

### 2.2 Immobilization and planning CT

Patients were immobilized in the supine position using Head & Neck Vac‐Lok Cushion, a thermoplastic head mask and the Uni‐frame Patient Positioning System (CIVCO, IA, USA) for the head and neck area, ESS‐15 (Engineering System Co., Ltd., Nagano, Japan) for the thoracic area, and Body Support II and ESF‐19 (Engineering System Co., Ltd., Nagano, Japan). Planning CT images were acquired in the supine position with 5‐mm slices using a 64‐slice CT (SCENARIA, Hitachi, Ltd., Tokyo, Japan). Since the TomoTherapy system has a couch with a limited translation length, two CT scans (head‐first position and feet‐first position) were performed for all patients. The head‐first position covered the range between the patients’ cranial vertex and the middle of the femurs, and the feet‐first position covered the range between the patients’ toes and the middle of the femurs.

### 2.3 Contouring

Contouring was performed with Pinnacle^3^ (Philips Medical System, Eindhoven, Netherlands). We created the following structures: clinical target volume (CTV), planning target volume 1 (PTV1), and PTV2. CTV consisted of an external body contour of the whole body. PTV1 consisted of CTV minus a 5‐mm margin under the skin surface. PTV2 consisted of CTV plus a 5‐ or 10‐mm (5/10 mm = 6 patients/7 patients) air margin. PTV1 and PTV2 were based on a previous report.^15^ The planning CT images and contours were exported to the TomoTherapy planning station (TomoHDA ver. 5.1.0.4, Accuray, CA, USA) for treatment planning.

### 2.4 Treatment planning and plan evaluation

Treatment planning was performed on the TomoTherapy planning station. The prescription was 4 Gy to cover 85% of the volume of the PTV1, and the dose per fraction was 2 Gy. We did not use dose constraints for OARs, as we do in our clinical routine for low‐dose TBI.

For the TomoDirect plans, we generated plans with 2–12 ports with approximately equally spaced angles^1^; in all, 11 TomoDirect plans were created for each patient. The detailed beam angles of each TomoDirect plan are shown in Table 2. The parameters of the modulation factor (MF), field width (FW), and pitch were 2.0, 5.0 cm, and 0.500, respectively. For the TomoHelical plans, the parameters of MF, FW, and pitch were 2.0, 5.0 cm, and 0.397, respectively. These parameters are default values for TBI with TomoTherapy in our institute. We also generated plans with a pitch of 0.430, which was the value previously used for comparison with plans with a pitch of 0.397. We used Dynamic jaw mode and a fine dose calculation grid for the final dose calculation process for both plans.

**Table 2:** Beam angles in TomoDirect plans

Number of ports	Beam angles (°)
2	90, 270
3	0, 120, 240
4	0, 90, 180, 270
5	0, 72, 144, 216, 288
6	0, 60, 120, 180, 240, 300
7	0, 51, 103, 154, 206, 257, 309
8	0, 45, 90, 135, 180, 225, 270, 315
9	0, 40, 80, 120, 160, 200, 240, 280, 320
10	0, 36, 72, 108, 144, 180, 216, 252, 288, 324
11	0, 32, 65, 98, 131, 164, 197, 230, 263, 296, 328
12	0, 30, 60, 90, 120, 150, 180, 210, 240, 270, 300, 330

To compare these treatment plans, D2 (near‐maximum dose), D98 (near‐minimum dose), D50 (median dose), and the homogeneity index (HI)^19^ of the PTV1 and the beam‐on time were evaluated. All the dose evaluations were performed on the PTV1. HI was defined by the following equation: HI = (D2–D98)/D50. An ideal value is equal to zero.^19^ According to the American College of Radiology (ACR) and American Society for Radiation Oncology (ASTRO) practice guideline,^8^ dose inhomogeneity should be maintained within ±10%. Therefore, we defined our criteria as follows: D2 should not exceed +10% of the prescribed dose (4.4 Gy) and D98 should not be below −10% of the prescribed dose (3.6 Gy) for all plans in this study.

Although we had two CT data sets because of the limitation of the couch motion with TomoTherapy, we used CT images for the head‐first position only for all treatment planning in this study.

### 2.5 Statistical analysis

We used the Wilcoxon signed‐rank test to compare the plans with a pitch of 0.397 with those with a pitch of 0.430. The Wilcoxon signed‐rank test was also performed to compare the dose evaluation indices between each of the 11 TomoDirect plans and the TomoHelical plan. Bonferroni correction was applied for multiple comparisons, and statistical significance was defined as *P* < 0.05/11 = 0.0045. We used JMP Pro 13 (SAS Institute Inc., Charlotte, NC, USA) for statistical analysis.

## 3 RESULTS

The D2 and D50 values for the pitch of 0.397 were statistically significantly worse than those for the pitch of 0.430 (D2; 4.16 Gy (4.12–4.21) *P* < 0.0002, D50; 4.07 Gy (4.06–4.08) *P* < 0.0002). However, the D98 and HI values for the pitch of 0.397 showed statistically significantly improvement over the pitch of 0.430 (D98; 3.81 Gy (3.75–3.88) *P* < 0.0002, HI; 0.087 (0.064–0.103) *P* < 0.0024). The pitch of 0.397 provided a more homogeneous dose distribution than the pitch of 0.430. Therefore, we used the data for the pitch of 0.397 for comparisons between the TomoDirect plans and TomoHelical plans.

**Figure 1:**
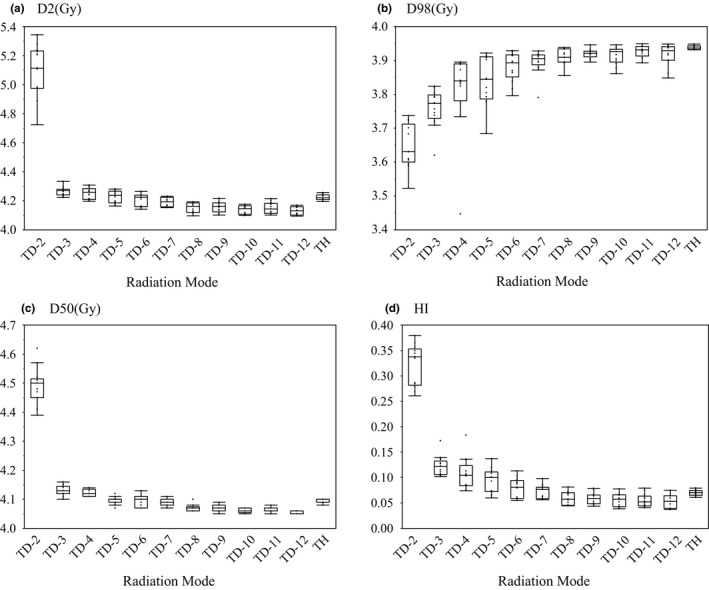
Dose evaluation index results for each port in the TomoDirect plans and for the TomoHelical plans. (a) D2, (b) D98, (c) D50, (d) HI. D2: the dose received by 2% of the volume; D98: the dose received by 98% of the volume; D50: median dose; HI: homogeneity index = (D2–D98)/D50; TD‐n: TomoDirect n‐port (n = 2–12); TH: TomoHelical.

Table 3 and Fig. 1 show the dose evaluation index results for each TomoDirect plan with 2–12 ports and the TomoHelical plans. All of the dose evaluation indices of the TomoDirect plans had a tendency to become better as the number of ports increased, but in the TomoDirect 2‐port plan, the D2 values ranged from 4.72 to 5.11 Gy, and the D98 values in two patients were less than 3.6 Gy. Also, in the TomoDirect 4‐port plan, the D98 value in one patient was 3.44 Gy. Thus, these plans did not meet the criteria of this study.

**Table 3:** Dose evaluation indices of TomoDirect and TomoHelical plans: median (range)

Radiation mode	D2 (Gy)	*P*‐value^a^	D98 (Gy)	*P*‐value^a^	D50 (Gy)	*P*‐value^a^	HI	*P*‐value^a^
TD‐2	5.11 (4.72–5.34)	0.0002*	3.63 (3.52–3.73)	0.0002*	4.50 (4.39–4.62)	0.0002*	0.337 (0.261–0.379)	0.0002*
TD‐3	4.27 (4.22–4.33)	0.0002*	3.77 (3.62–3.82)	0.0002*	4.13 (4.10–4.16)	0.0002*	0.122 (0.102–0.172)	0.0002*
TD‐4	4.26 (4.20–4.31)	0.0037*	3.84 (3.44–3.90)	0.0002*	4.12 (4.11–4.14)	0.0002*	0.104 (0.074–0.218)	0.0002*
TD‐5	4.24 (4.16–4.28)	0.9060	3.84 (3.68–3.92)	0.0002*	4.10 (4.07–4.12)	0.4492	0.100 (0.060–0.137)	0.0012*
TD‐6	4.22 (4.14–4.27)	0.0598	3.89 (3.80–3.93)	0.0002*	4.10 (4.07–4.13)	0.9355	0.081 (0.055–0.113)	0.0574
TD‐7	4.20 (4.15–4.23)	0.0012*	3.91 (3.79–3.93)	0.0002*	4.09 (4.07–4.11)	0.2168	0.077 (0.056–0.098)	0.6848
TD‐8	4.16 (4.10–4.19)	0.0002*	3.91 (3.86–3.94)	0.0002*	4.07 (4.06–4.10)	0.0005*	0.057 (0.044–0.082)	0.0007*
TD‐9	4.16 (4.10–4.22)	0.0002*	3.92 (3.90–3.95)	0.0005*	4.07 (4.05–4.09)	0.0002*	0.058 (0.043–0.079)	0.0005*
TD‐10	4.15 (4.10–4.18)	0.0002*	3.93 (3.86–3.95)	0.0005*	4.06 (4.05–4.07)	0.0002*	0.057 (0.039–0.078)	0.0002*
TD‐11	4.14 (4.10–4.22)	0.0002*	3.93 (3.89–3.95)	0.0205	4.06 (4.05–4.08)	0.0002*	0.052 (0.040–0.079)	0.0005*
TD‐12	4.13 (4.10–4.17)	0.0002*	3.93 (3.85–3.95)	0.0156	4.06 (4.05–4.06)	0.0002*	0.053 (0.037–0.075)	0.0002*
TH	4.22 (4.20–4.26)	–	3.94 (3.93‐3.95)	–	4.09 (4.08–4.10)	–	0.070 (0.061–0.080)	–

D2: the dose received by 2% of the volume; D98: the dose received by 98% of the volume; D50: median dose; HI: homogeneity index = (D2–D98)/D50; TD‐n: TomoDirect n‐port (n = 2–12); TH: TomoHelical (pitch = 0.397).

a Comparison between each TomoDirect plan with 2–12 ports and the TomoHelical plans.

*P < 0.0045 (Bonferroni correction).

**Figure 2:**
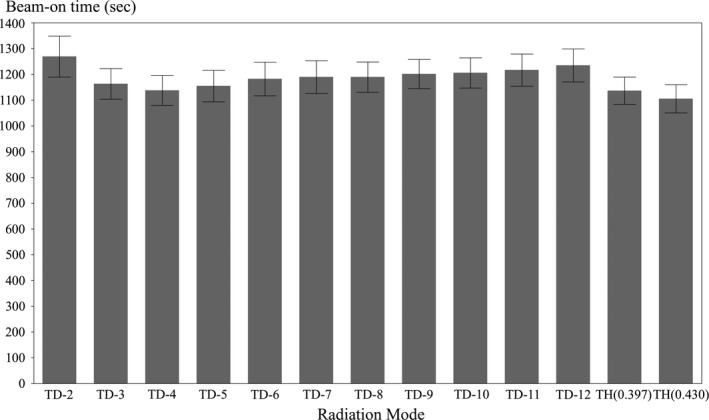
Beam‐on time results for each port in the TomoDirect plans and for the TomoHelical plans. TD‐n: TomoDirect n‐port (n = 2–12); TH: TomoHelical.

Table 4 and Fig. 2 show the beam‐on time of each TomoDirect plan with 2–12 ports and the TomoHelical plans. TomoDirect with two ports required the longest beam‐on time. Conversely, the TomoHelical plan with a pitch of 0.430 required the shortest beam‐on time. The beam‐on time in the TomoDirect plans with four ports or more had a tendency to become longer as the number of ports increased.

**Table 4:** Beam‐on time of TomoDirect and TomoHelical plans (mean ± SD)

Radiation mode	Beam‐on time (s)
TD‐2	1269 ± 79.6
TD‐3	1163 ± 59.5
TD‐4	1137 ± 58.1
TD‐5	1154 ± 61.3
TD‐6	1182 ± 65.2
TD‐7	1189 ± 63.4
TD‐8	1189 ± 58.7
TD‐9	1201 ± 56.8
TD‐10	1206 ± 58.6
TD‐11	1217 ± 62.5
TD‐12	1235 ± 64.0
TH (pitch = 0.397)	1136 ± 58.8
TH (pitch = 0.430)	1105 ± 54.8

TD‐n: TomoDirect n‐port (n = 2–12); TH: TomoHelical.

**Figure 3:**
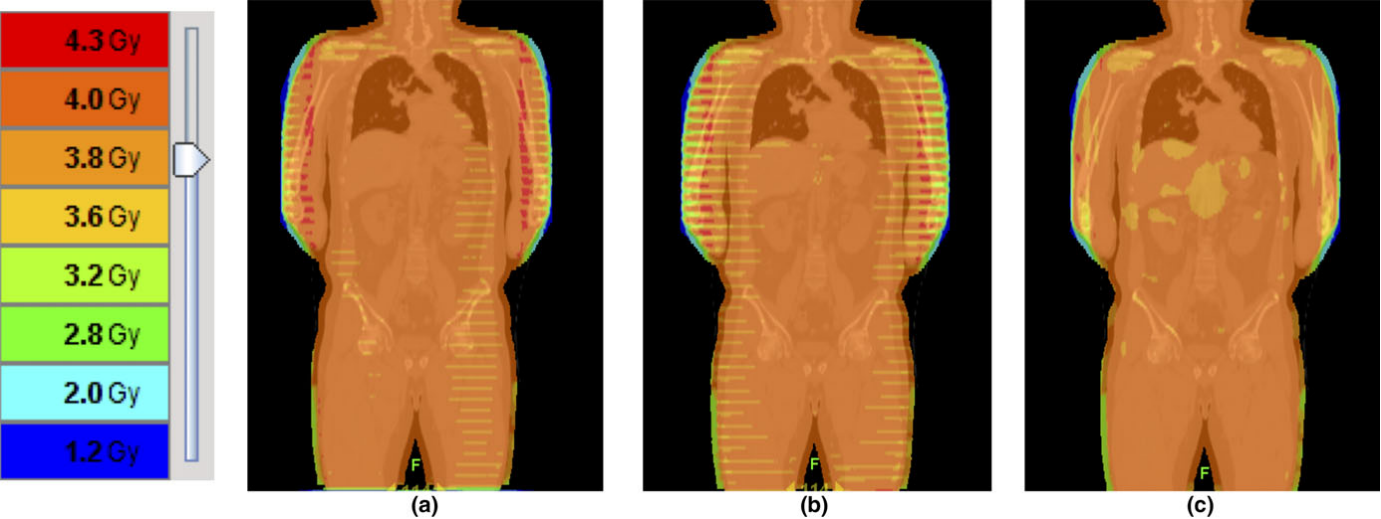
Dose distributions in the same patient. (a) TomoHelical plan (pitch = 0.397), (b) TomoHelical plan (pitch = 0.430), (c) TomoDirect plan (12‐port). The angle distributions in the TomoDirect plan were 0°, 30°, 60°, 90°, 120°, 150°, 180°, 210°, 240°, 270°, 300°, and 330°.

Figure 3 shows the differences in the dose distribution between the 12‐port plan of TomoDirect plan and the TomoHelical plans (pitch = 0.397 and 0.430) in the same patient. In the TomoHelical plans, dose variation patterns known as the thread effect were observed, especially in both arms of the patient. These variation patterns were conspicuous at the pitch of 0.430. By contrast, such variation patterns were not observed in the TomoDirect plan.

### 3.1 Comparison between TomoDirect and TomoHelical plans

Table 3 shows the results of the comparisons of D2, D98, D50, and HI between the TomoDirect plans with 2–12 ports and the TomoHelical plan. The D2 values of the TomoDirect plans with four ports or fewer were statistically significantly worse than that of the TomoHelical plan; however, the values for the TomoDirect plans with seven ports or more were statistically significantly better than that of the TomoHelical plan. The D98 values of the TomoDirect plans with ten ports or fewer were statistically significantly worse than that of the TomoHelical plan, and there were no statistically significant differences between the TomoDirect plans with 11 ports or more and the TomoHelical plan. The D50 values of the TomoDirect plans with four ports or fewer were statistically significantly worse than that of the TomoHelical plan, but those of the TomoDirect plans with eight ports or more showed statistically significant improvement over the TomoHelical plan. In terms of HI, the TomoDirect plans with five ports or fewer had statistically significantly worse values than the TomoHelical plan, but the TomoDirect plans with eight ports or more showed statistically significant improvement over the TomoHelical plan.

## 4 DISCUSSION

We investigated the relationships between the number of ports and the dose evaluation indices in TBI using 2‐ to 12‐port TomoDirect plans and compared the results with that of the TomoHelical plan. We found that TomoDirect had a dosimetric advantage over TomoHelical; however, the beam‐on time was longer in TomoDirect. We should consider prolonging the treatment time with TomoDirect when considering clinical application.

In the TomoDirect 2‐port plan, D2 (the near‐maximum dose) was 5.11 Gy (4.72–5.34 Gy), and the minimum value of D98 (the near‐minimum dose) in 13 patients was 3.52 Gy. Considering the practice guideline^8^ and the criteria of this study, the TomoDirect 2‐port plan may be unacceptable for clinical use. Furthermore, in the TomoDirect 4‐port plan, the minimum value of D98 was 3.44 Gy; thus, this plan would also be unacceptable.

We found statistically significant improvements of D2, D50, and HI in the TomoDirect plans compared with TomoHelical plan. These differences may be caused by the thread effect in TomoHelical mode, which is known to be a dose‐variation pattern that manifests as a ripple, which is the result of helical beam junctioning.^20^, ^21^, ^22^ Takahashi et al.^22^ investigated dose heterogeneity with TomoHelical at various skeletal regions due to the thread effect in total marrow irradiation, and found that the maximum left‐to‐right arm distance strongly correlated with the thread effect and resulted in dose heterogeneity, particularly in the bones of the arm. They discussed the absolute importance of homogeneous dose delivery even for the extremities, because relapses of hematological malignancies from the extremities have been reported.^23^, ^24^ They also mentioned that their findings were applicable for TBI and total skin irradiation. In this study, we observed dose variation patterns caused by the thread effect in the both arms when using the TomoHelical plans shown in Fig. 3. The pitch of 0.430 (pitch = 0.86/n, n: integer) was reported to minimize ripples^20^; however, we observed these variation patterns to be more conspicuous, especially in the both arms than with a pitch of 0.397. Similar results were reported in a previous study.^22^ The pitch of 0.397 was reported to minimize ripples in the setting of an off‐axis distance = 20 cm and FW = 5.0 cm.^21^ This study also showed a statistically significant improvement of dose homogeneity with a pitch of 0.397. We consider the beam‐on time with the pitch of 0.397 to be acceptable because it was prolonged by about 30 s compared with the pitch of 0.430. Therefore, although there have been several reports^3^, ^6^, ^15^, ^16^ in which a pitch of 0.430 was used, we suggest that a pitch of 0.397 is better pitch for TBI with TomoHelical. By contrast, we did not observe these variation patterns with the TomoDirect plans. There were several heterogeneous regions in the abdomen when using the TomoDirect plans compared with the TomoHelical plans, however, the TomoDirect plans showed statistically significant improvement in dose homogeneity as shown in Table 3. The dose heterogeneity induced by the thread effect might have more impact on low‐dose TBI than myeloablative TBI because of the low number of fractions. Thus, we consider that TomoDirect plans with more than eight ports can provide significant improvement in dose homogeneity compared with TomoHelical plans. However, dosimetric measurements and comparisons of the thread effect in TBI between TomoHelical and TomoDirect plans are needed.

This study has two limitations. First, we only evaluated physical values at the treatment planning station. Our results suggest that low‐dose TBI with TomoDirect would not require 12 ports, the maximum number of ports in TomoDirect, because some dose evaluation indices for TomoDirect using fewer than 12 ports showed significant improvement over TomoHelical. Reducing the number of ports lead to a decrease in the treatment time, including the beam‐on time; thus, the investigation of dose verification and measurements in low‐dose TBI using TomoDirect with fewer than 12 ports is required in order to verify its validity. Second, this study only investigated patients undergoing low‐dose TBI. We usually do not define OARs such as eyes, lungs, liver, and kidneys and their respective dose constraints in our clinical routine for low‐dose TBI; however, we should consider limiting the dose for those OARs in myeloablative TBI. The difference in the delivery technique between TomoDirect and TomoHelical may lead to differences in PTV coverage and in the capability to spare capability of OARs sparing. Consequently, further investigation of the relationships between the number of ports and the dose evaluation indices for both the target volume and OARs using TomoDirect, and the relationships of the dose evaluation indices to TomoHelical values, are needed to evaluate of these methods in myeloablative TBI.

## 5 CONCLUSIONS

We investigated the relationships between the number of ports and the dose evaluation indices in low‐dose TBI using TomoDirect with 2–12 ports and compared the results to those obtained with TomoHelical. Statistically significant improvements of D2, D50, and HI but not D98 were found with the TomoDirect plans. Further investigations including dose verification and measurements in low‐dose TBI using TomoDirect with fewer than 12 ports are necessary.

## CONFLICT OF INTEREST

The authors declare no conflict of interest.
